# Fine scale analysis of malaria incidence in under-5: hierarchical Bayesian spatio-temporal modelling of routinely collected malaria data between 2012–2018 in Cameroon

**DOI:** 10.1038/s41598-021-90997-8

**Published:** 2021-06-01

**Authors:** Celestin Danwang, Élie Khalil, Dorothy Achu, Marcelin Ateba, Moïse Abomabo, Jacob Souopgui, Mathilde De Keukeleire, Annie Robert

**Affiliations:** 1 Epidemiology and Biostatistics Unit, Institut de Recherche Expérimentale et Clinique, Université catholique de Louvain, Box: B1.30.13, Brussels, Belgium; 2 National Malaria Control Program, Ministry of Public Health, Yaoundé, Cameroon; 3 Department of Molecular Biology, Institute of Biology and Molecular Medicine, Universite Libre de Bruxelles, Gosselies, Belgium

**Keywords:** Malaria, Epidemiology

## Abstract

The current study aims to provide a fine-scale spatiotemporal estimate of malaria incidence among Cameroonian under-5, and to determine its associated environmental factors, to set up preventive interventions that are adapted to each health district of Cameroon. Routine data on symptomatic malaria in children under-5 collected in health facilities, between 2012 and 2018 were used. The trend of malaria cases was assessed by the Mann–Kendall (M–K) test. A time series decomposition was applied to malaria incidence to extract the seasonal component. Malaria risk was estimated by the standardised incidence ratio (SIR) and smoothed by a hierarchical Bayesian spatiotemporal model. In total, 4,052,216 cases of malaria were diagnosed between 2012 and 2018. There was a gradual increase per year, from 369,178 in 2012 to 652,661 in 2018. After adjusting the data for completeness, the national incidence ranged from 489‰ in 2012 to 603‰ in 2018, with an upward trend (M–K test p-value < 0.001). At the regional level, an upward trend was observed in Adamaoua, Centre without Yaoundé, East, and South regions. There was a positive spatial autocorrelation of the number of malaria incident-cases per district per year as suggested by the Moran’s I test (statistic range between 0.11 and 0.53). The crude SIR showed a heterogeneous malaria risk with values ranging from 0.00 to 8.90, meaning that some health districts have a risk 8.9 times higher than the national annual level. The incidence and risk of malaria among under-5 in Cameroon are heterogeneous and vary significantly across health districts and seasons. It is crucial to adapt malaria prevention measures to the specificities of each health district, in order to reduce its burden in health districts where the trend is upward.

## Introduction

Significant efforts have been dedicated to the fight against malaria worldwide over the last two decades. These efforts have contributed to a reduction in the incidence, and mortality related to malaria since 2010^1–4^. Indeed, the World Health Organisation (WHO) estimates that the incidence of malaria has decreased worldwide from 71 to 57 per thousand population at risk between 2010 and 2018, with South-East Asia registering a 70% reduction, compared to a 22% reduction in sub-Saharan Africa^4^. Although all age groups are susceptible to malaria, the disease disproportionately affects children under five years (under-5) whose immune system is less developed, and can result in many complications such as anaemia, acute kidney injury, or death; in 2018, 67% of malaria-related deaths were in the under-5^4–6^.

To stay on track with the progress made between 2000 and 2010, Cameroon has made the fight against malaria a public health priority, particularly among children under-5 and pregnant women^7–9^. Hence, since 2010, the country has significantly increased the number of interventions to reduce the burden of malaria. These include free intermittent preventive treatment of malaria in pregnant women, free treatment of malaria in children under-5, distribution of mosquito bed nets throughout the country, training of community health workers to diagnose and treat malaria, and digitisation of the national malaria data collection system. The latter has provided accurate data to estimate the incidence of malaria in different areas of the country and to see the specificities of each region in terms of malaria burden.

Recent advances in spatiotemporal epidemiology have made it possible to provide an area-specific estimate that can guide policy-makers in tailoring interventions according to the specificity of each geographical unit^2,3,10^. This initiative, led by the Malaria Atlas Project (MAP) team, has drawn attention on the need for fine-scale disaggregated estimates to significantly reduce the global malaria burden^3,10,11^.

In Cameroon, given the variation in vegetation and climatic conditions in each region of the country, the biotope of the malaria vector (*Anopheles*) varies greatly by region and by the period of the year (rainy or dry season)^8,12–15^; suggesting also a need for a fine-scale temporal analysis for decision-making rather than aggregated country-level data that shadows the epidemiological specificity of each area.

This study aimed at providing a fine-scale analysis of symptomatic malaria incidence in under-5, and at assessing the time trend and seasonality of malaria between 2012 and 2018, and their association with environmental factors.

## Methods

### Study setting

Cameroon is located in Central Africa (2–13° N) and characterized by three different climatic zones^16^. The equatorial zone (2–9° N) is marked by an average annual temperature of about 25 °C and the presence of rainfall throughout the year, favouring the year-round growth of female Anopheles, the malaria vectors. The Sudanian zone, lying between 7–10° N, is typified by less abundant rainfall with a dry season lasting 5–6 months and an average annual temperature of about 22 °C. The Sudano-Sahelian zone, lying between 10–13° N, is the driest and is characterised by a shorter annual rainy season of about 2–3 months^8,16^.

Between 2012 and 2018, Cameroon's population grew from 20.52 million to 23.80 million, with 44.6% of the population aged less than 15 years^17^. The country is administratively divided into 10 regions (North-West, Adamaoua, East, South, Far North, Centre, Littoral, North, West and South-West). For consistency in our analysis, the two largest cities in the country, Douala (located in the Littoral region) and Yaoundé (in the Centre region), have been considered as two separate regions, giving a total number of 12 regions in our analysis. The regions located in the equatorial zone are South, Centre, Littoral, East, and South-West, while those in the Sudanian zone are West and North-West. The Sudano-Sahelian zone contains the regions of Adamaoua, North and Far North. The entomological inoculation rate of malaria varies by region and is about 6.0–20.0 ib/p/m in the Far North, 24.5–60.0 ib/p/m in the North, 100 ib/p/yr in Adamaoua, 2.2–11.0 ib/p/yr in West and North-West, 0.7–1.4 ib/p/m in South, Centre, Littoral, East, and South-West, and 0–90.0 ib/p/yr in Douala and Yaoundé^8^.

The Cameroonian health system has a pyramidal structure with the operational level (health district) at its base. The division into health districts does not overlap the boundaries of the regions. In 2018, the country had 189 health districts (see Supplemental Fig. S1 online).

### Data source

The data on malaria in children used in this study are those compiled by the National Malaria Control Program (NMCP). Cameroon has an electronic malaria data collection system (DHIS-2). Data from all public and private health facilities in the country are entered monthly into a single electronic database. This allows a pooling at the level of health areas, health districts, or regions. The health-seeking behaviour was obtained from the Demographic Health Survey (DHS) programme and Multiple Indicator Cluster Survey (MICS). The environmental data, i.e., temperature, rainfall, and vegetation from 2012 to 2018 were retrieved from official sources presented in the appendix (see Supplemental Table S1 online).

### Malaria case definition and estimation of the incidence rate

As these data are collected by health facilities, a confirmed case of malaria was defined as the presence in a symptomatic patient (fever, headache or other malaria-related symptoms) of a positive malaria test (either a rapid diagnostic test or a blood smear)^18^. To estimate the incident cases number in each health district, the raw number of reported cases was adjusted according to the method described by Cibulskis et al.^19^ to provide a more accurate estimate of incident cases as follow:

$${\text{M}} = {\text{Mu}} + {\text{Ml}},\;{\text{with}}\;{\text{Mu}} = {{\left( {{\text{C}} + {\text{sU}}} \right)} / {{\text{rp}}}}\;{\text{and}}\;{\text{Ml}} = {{\left( {{\text{C}} + {\text{sU}}} \right)\left( {1 - {\text{n}}} \right)} / {{\text{rp}}}}$$

where M is the annual number of incident-cases for the target area, C is the annual reported number of confirmed malaria cases, U is the annual reported number of unconfirmed cases, s is the slide positivity rate or proportion of RDTs that gives positive result, r is the completeness of health-facility reports, p is the proportion of the population with fever that uses health facilities covered by the public health-facility reporting system, and n is the proportion of fever cases that do not seek treatment. All data except p and n (which were retrieved from DHS and MICS) were obtained from the NMCP. This adjustment is made because in sub-Saharan African countries, not all malaria cases occurring in the communities are taken to hospitals for treatment. Indeed, some cases will resort to traditional medicine, or go directly to the pharmacy to obtain a medicine or seek advice from health personnel without going to the health facilities. Thus, the number of registered cases should be adjusted as described above to give a figure close to the reality that would have been observed if all cases of malaria occurring in the community were registered in the health facility databases.

The adjusted number of incident-cases was then divided by the total number of children at risk in each health district to obtain the incidence rate by district by year. The number of children at risk was obtained from estimates made by the Cameroon National Institute of Statistics after the 2005 census.

As no information on the species responsible for malaria was provided, all cases were considered to be due to *Plasmodium* *falciparum*^20^.

### Standardized incidence ratio

The crude annual standardised incidence ratio (SIR) was calculated as

$${\text{SIR}}_{{{\text{it}}}} = \xi_{{{\text{it}}}} /\zeta_{{{\text{it}}}} ,$$

where, ξ_it_ is the observed number of cases of malaria in the district i during the year t, ζ_it_ the expected number of cases of malaria in the district i during the year t, and SIR_it_ the standardized incidence ratio of malaria in the district i during the year t.

The expected number of cases in each district was obtained by multiplying the annual national malaria incidence rate by the population size of each district.

The SIR was computed to give a crude estimate of the risk of malaria at the health district level, prior to modelling.

### Time series decomposition

The incidence rate per region per month was obtained by dividing the number of reported cases adjusted for completeness by the population at risk in the region. The Mann–Kendall trend test (M–K) was performed to assess the trend in malaria incidence in each region of the country. A seasonal trend decomposition procedure based on LOESS (STL) was applied as proposed by Cleveland and colleagues^21^, yielding to a trend component of the log-transform malaria incidence rate, a seasonal component, and a remainder. Prior to the STL decomposition, the monthly incidence rate was considered as a time series.

As the incidence of malaria varies according to the period of the year, we have decomposed the incidence rate to extract the seasonal variation, and the linear trend observed in each region. This was done to highlight the particularity of malaria seasonality in each region of the country and to identify the linear trend. The MK test was performed to objectively assess whether there was a monotonic trend in the incidence of malaria observed after the STL decomposition.

### Bayesian spatiotemporal modelling

#### Selection of explanatory variables

Covariates known to be associated with malaria occurrence, namely temperature, rainfall and vegetation, were retrieved (see Supplemental Table S1 online). Four lags (i.e., the minimum temperature the month before representing minimum temperature lag 1, the minimum temperature two months before representing minimum temperature lag 2) were generated for each covariate. Pearson’s correlation coefficient was used to assess the correlation between factors related to the same covariate (e.g., minimum temperature: mean, mean lag 1, mean lag 2, mean lag 3, mean lag 4 as well as median and median lag 1 to 4) and malaria count. The factor with the highest correlation was selected for modelling. The final set of covariates included the median value of the maximum temperature lag 1. The mean rainfall lag 1, and the mean normalized difference vegetation index (NDVI) (see Supplemental Fig. S2 online).

#### Spatial and temporal dependency

A binary spatial and temporal neighbourhood matrix was used to integrate the dependency as described by Lee and colleagues^22^. For the spatial neighbourhood matrix, 1 represents two health districts sharing a common boundary and 0 otherwise. While for the temporal neighbourhood matrix, 0 if the time periods are not one unit apart, and 1 if there are^22^.

The spatial dependency was assessed by the Moran’s I test, and a *P*-value < 0.05 led to conclude to spatial autocorrelation. A Moran’s I statistic close to one was indicative of a strong positive autocorrelation, while a value close to − 1 indicated a negative autocorrelation^22^. The spatial dependency was assessed on the residual of a first cross-sectional model (negative-binomial log-linear model without spatial or temporal term) with malaria counts as outcome variable and environmental variables (the maximum temperature lag 1, The mean rainfall lag 1, and the mean normalized difference vegetation index) as independent variables.

#### Model building

Hierarchical Bayesian spatiotemporal models using integrated nested Laplace approximations implemented in the R-INLA package were used to smooth the estimated malaria risk, assuming a negative binomial distribution for malaria counts^23,24^.

The model was constructed with the above-mentioned covariates, to which were added a spatially unstructured random effect term, a spatially structured conditional autoregressive term (CAR), a first-order random walk-correlated time term, and a time–space interaction term^24^.

The initial model (frailty model) was a simple random-effect model with only the spatially-unstructured heterogeneity component without covariate, as describe by DiMaggio et al.^25^. Subsequently convolutional models were built as follow: the first convolutional model (model 1) was constructed by adding to the frailty model (spatially unstructured term noted ν_i_) a spatially structured conditional autoregression term (υ_i_) giving the following model: *y*_*i*_ = α + υ_i_ + ν_i_. The other models were structured as follow.

Model 2: convolutional model one with uncorrelated time, model 3: convolutional model one with 1st order random walk correlated time, model 4: convolutional model one with 1st order random walk correlated time and uncorrelated time, model 5: convolutional model one with 1st order random walk correlated time and space–time interaction term, and model 6: convolutional model one with 1st order random walk correlated time, uncorrelated time, and space–time interaction term.

The best model was chosen based on the lowest Deviance Information Criterion (DIC), then covariates were added to give the final full model. In space–time Bayesian models, the exceedance probability represents the probability of occurrence of the outcome above a given value. This value was set to one in our analysis.

Unless indicated, a *P*-value lower than 0.05 was considered as a statistically significant result. R software (version 4.0.2) was used for the analyses.

### Ethics declarations

Ethical approval (No:2020/17MAR/161) was obtained from the Ethics Committee of UCLouvain before the beginning of this study. The data were anonymised and aggregated at the health district level without any personal patient information. All methods were performed in accordance with the relevant guidelines and regulations.

## Results

### Malaria incidence and seasonality

**Figure 1:**
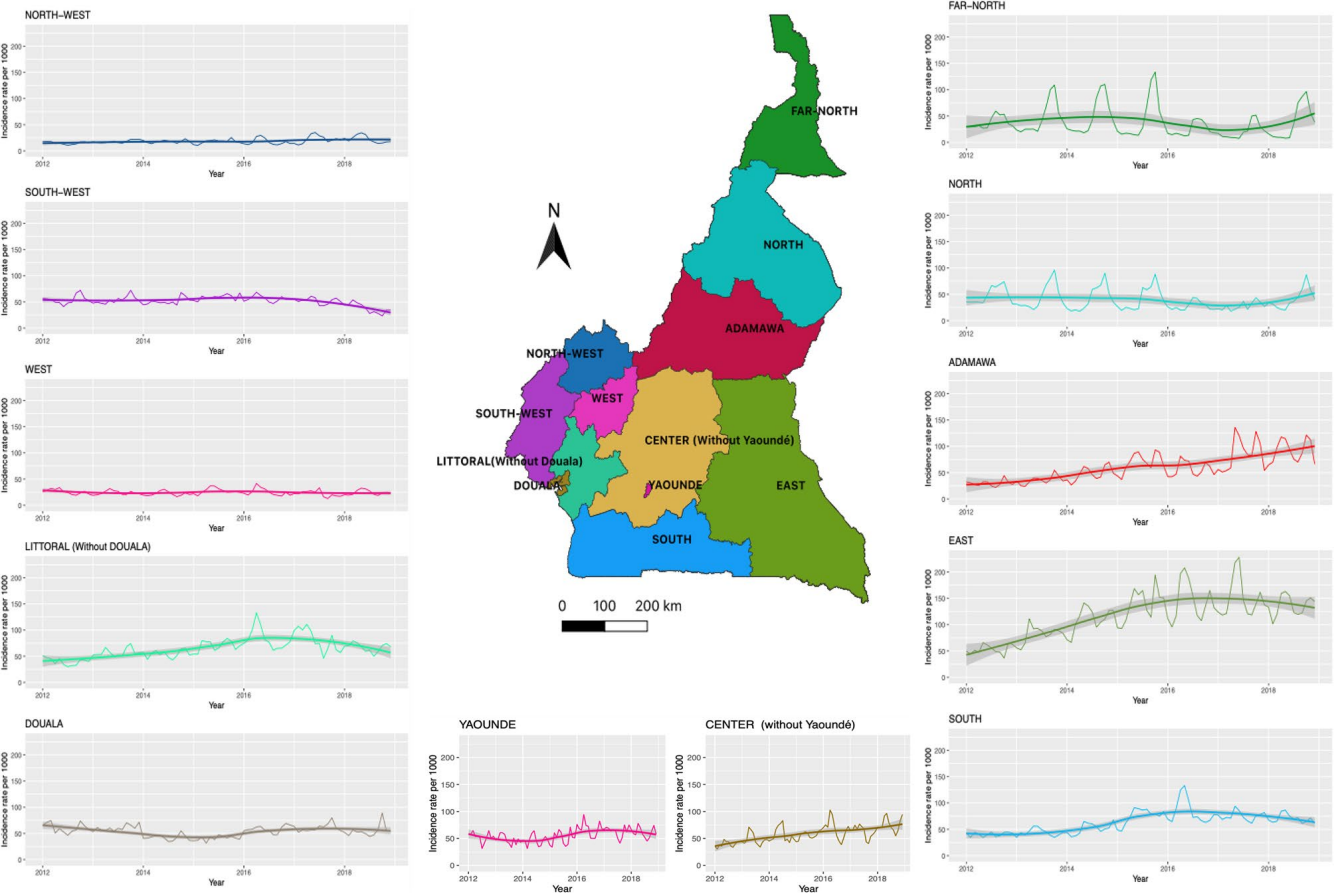
Malaria incidence rate in under-fives by region between 2012–2018. Each panel represents the trend in U-5 malaria incidence in one of the country's regions between the years 2012 and 2018. The two largest cities in the country (Douala and Yaoundé) are shown separately, outside the regions where they are located. The names of the regions are mentioned at the top of each panel and the map in the middle gives the geographical location of each region. The map was generated with QGIS version 3.4.8 (URL: https://qgis.org/en/site), and the graphics with the ggplot2 package of R software version 4.0.2 (URL: https://cran.r-project.org/web/packages/ggplot2/index.html).

Between 2012 and 2018, 4,052,216 cases of malaria were diagnosed in under-5 in Cameroon, either by blood smear or RDT. With a progressive increase per year from 369,178 in 2012 to 652,661 in 2018. The equatorial zone recorded the highest number of incident cases (2,134,187 cases), followed by the Sudano-Sahelian zone (1,403,994 cases) and the Sudanian zone (514,036 cases). After adjusting the raw data for completeness, and computing the incidence rate, there was an upward trend in incidence of malaria at the national level (p-value M–K: < 0.001). However, four of the twelve regions, namely East (M–K p-value: 0.04), South (M–K p-value: 0.07), Centre without Yaoundé (M–K p-value: 0.02), Adamaoua (M–K p-value: 0.007), showed an upward trend, while the South-West (M–K p-value: 0.37), West (M–K p-value: 0.76), Douala-Littoral (M–K p-value: 0.76), Far North (M–K p-value: 0.22), North-West (M–K p-value: 0.13), Littoral [without Douala] (M–K p-value: 0.13), Yaoundé-Centre (M–K p-value: 0.37) had no monotonic trend, and North (M–K p-value: 0.04) a downward trend (see Fig. 1 and Supplemental Table S2 online). When looking at the incidence in the different climatic zones, only the equatorial zone showed a significant upward trend (p-value M–K < 0.001), while the Sudano-Sahelian zone and the Sudanian zone did not exhibit any monotonic trend.

**Figure 2:**
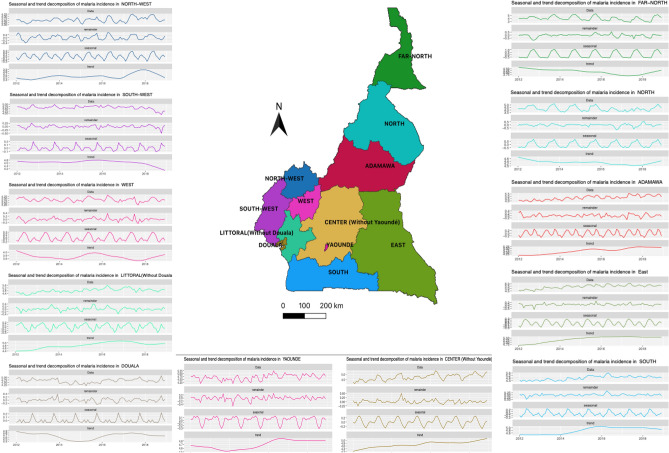
Seasonal-trend decomposition of malaria incidence by region between 2012–2018. Each panel represents the decomposition of malaria incident-cases recorded in health facilities among under-fives in a given region of Cameroon between the years 2012 and 2018. The two largest cities of the country (Douala and Yaoundé) are presented separately, outside the regions where they are located. The names of the regions are mentioned at the top of each panel and the map in the middle gives the geographical location of each region. On each graph, the first curve on the top (Data) represents the raw data, while the last one at the bottom (trend) represents the trend. The two middle curves represent from top to bottom: the remainder and the seasonal component of the seasonal-trend decomposition based on Loess. The map was generated with QGIS version 3.4.8 (URL: https://qgis.org/en/site), and the graphics with the ggplot2 package of R software version 4.0.2 (URL: https://cran.r-project.org/web/packages/ggplot2/index.html).

A seasonal pattern of malaria incidence rates, varying by region, was found on the seasonal-trend decomposition (Fig. 2). A positive correlation with rainfall the month before (r = 0.60), and negatively correlated with the maximum temperature the month before (r = − 0.44) and the number of malaria incident-cases was found (see Supplemental Fig. S2 online). At the national level, malaria incidence peaked annually between June and October (highest peak) and April and June (smallest peak) (Supplemental Fig. S3 online). At the regional level, the East, North, Far North, North-West, South, West, Yaoundé-Centre, Adamaoua and the Centre without Yaoundé had a seasonality marked by two annual peaks. The highest peak was between June and October in the North, Far North, Adamaoua and between February and June in the Centre without Yaoundé (Fig. 2).

### Standardized incidence ratio and spatial autocorrelation

**Figure 3:**
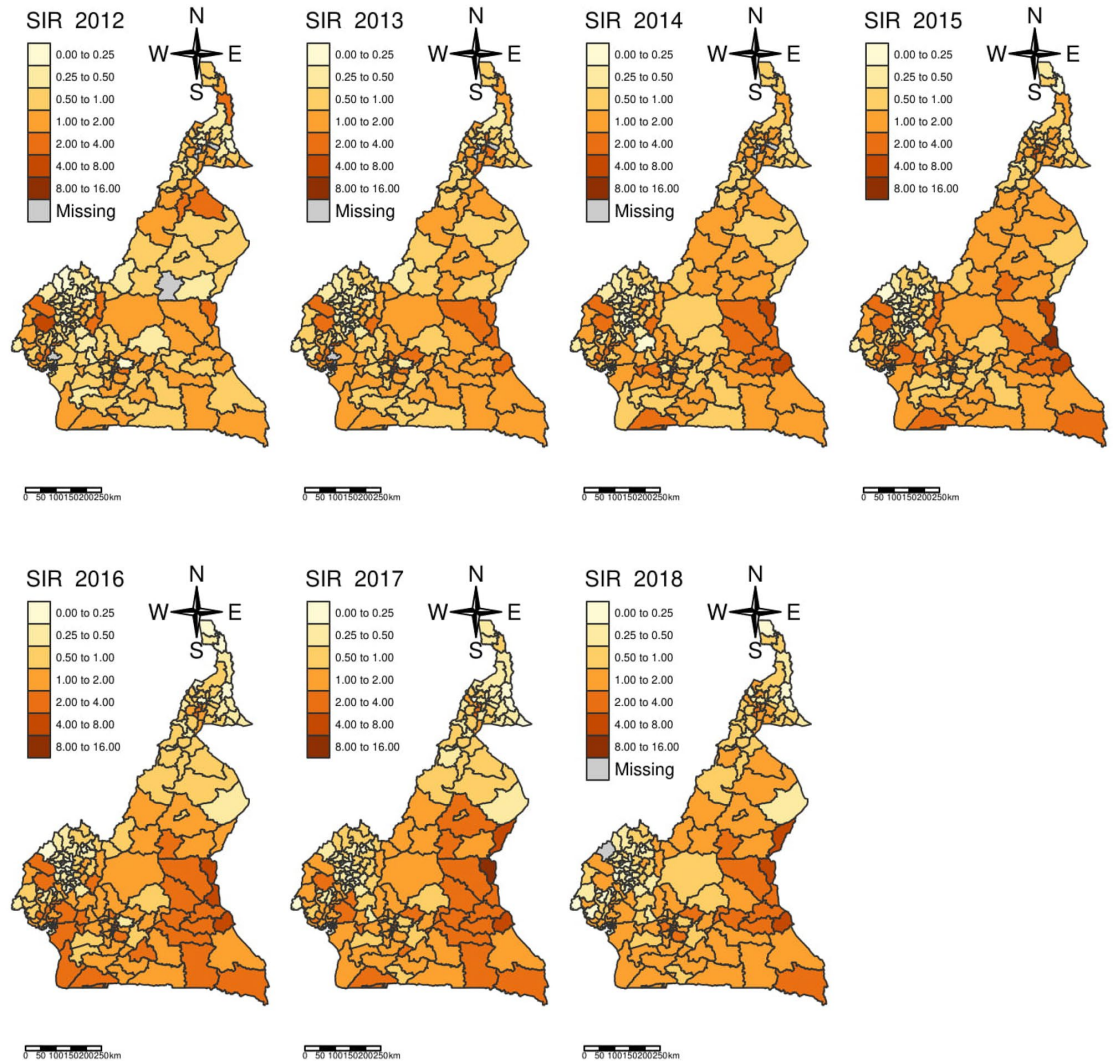
Map of malaria standardized incidence ratio in under-fives between 2012–2018. The SIRs are presented by year and by health districts for the years 2012 to 2018. The map was generated with tmap package of R software version 4.0.2 (URL: https://cran.r-project.org/web/packages/tmap/vignettes/tmap-getstarted.html).

The standardised incidence ratio at the health district level per year during the study period suggested a substantial heterogeneity in the risk of malaria among under-5 in the different regions of the country, and between districts within the same region. Some health districts had an annual risk 4–5 times higher than others in the same region (Fig. 3). This heterogeneity was found in every year of the time series (Fig. 3). The East region cumulated the health districts with the highest risk.

Concerning the spatial autocorrelation, the value of the Moran’s I test pointed to a positive spatial autocorrelation with values of the Moran statistics ranging from 0.11 to 0.53 (see Supplemental Table S3 online).

### Spatial and temporal modelling

The model with spatially unstructured term, the spatially structured conditional autoregression term and covariates had the lowest DIC (see Supplemental Table S4 online) and was kept as the more suitable model (see Supplemental Table S5 online). In this final model, spatially structured variance accounted for 84.5% of the total variance. The maps of the spatially structured conditional autoregression term of the final model with and without covariates are presented in the appendix (see Supplemental Fig. S4 online). The unstructured random effect term of the frailty and of the final model with covariates are shown on the map in appendix (see Supplemental Fig. S5 online) as well as their densities plots (see Supplemental Figs. S6–S10 online).

Results of the fixed effect model and the effect of each environmental covariate on malaria risk when all other covariates are held at zero are presented in the appendix (see Supplemental Table S5 online). The results presented are the point estimates with the uncertainty around the point estimates.

**Figure 4:**
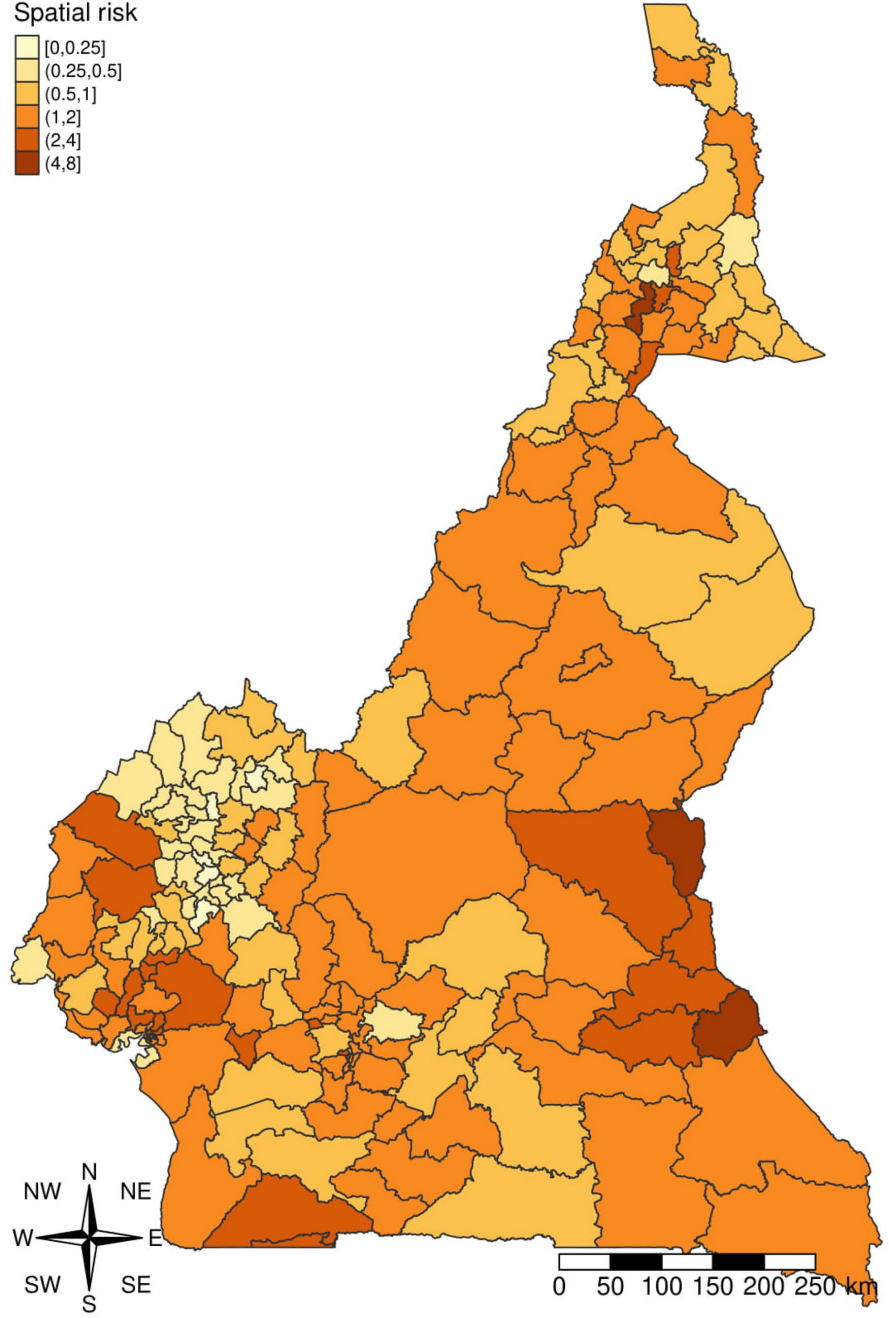
Modelled malaria risk map. The spatial risk map shown here is the result of the Bayesian model with the lowest DIC. The map was generated with tmap package of R software version 4.0.2 (URL: https://cran.r-project.org/web/packages/tmap/vignettes/tmap-getstarted.html).

The final spatial risk map shows an heterogenous risk of malaria across the country (Fig. 4). Two (Garou-Boulaï and Ndelélé) of the three health districts with the highest risk were in the East region while the third one was in the Far North region. Overall, the East region had the highest number of health districts with a risk of malaria greater than one (six health districts out of 189) (Fig. 4). The equatorial zone had more high-risk health districts as suggested by the model risk map, followed by the Sudano-Sahelian zone (Fig. 4). This indicated that the equatorial zone has the highest malaria burden compared to the others. The exceedance probability risk map (RR > 1) shows that all health districts located in the East region had a probability greater than 0.75 to have a malaria RR greater than 1 (see Supplemental Fig. S11 online).

## Discussion

The current analysis of reported cases of symptomatic malaria among children under-5 routinely collected by the country's health facilities in Cameroon shows that 4,052,216 cases of malaria have been diagnosed in this age group between 2012 and 2018, with an upward trend in the incidence rate over time at the national level. In addition, the results suggest a substantial variation in the malaria trend, seasonality across health districts and a different pattern of malaria risk across years. Furthermore, a strong positive correlation of the malaria incidence rate with temperature and rainfall was found.

Despite intensified efforts to reduce the burden of malaria in the under-5 in Cameroon, we found an upward trend in malaria incidence at the national level during the study period, as confirmed by the Mann–Kendall trend test. This is probably multi-factorial and related to the gradual improvement in both the country's malaria diagnostic capacity and the efficiency of the health reporting system, but also to the underuse of preventive measures.

Indeed, during the study period, the availability and accessibility of rapid diagnostic testing for malaria increased considerably^26^. With more community health workers who were trained to diagnose and treat malaria, the number of reported cases increased between 2016 and 2018. The reason being that cases that were previously undiagnosed because they lived in remote areas with poor access to health facilities, were now diagnosed, treated free of charge and reported in the national reporting system^26^.

Moreover, in Cameroon only 3–5% of the population is covered by health insurance, and due to lack of resources, parents are less likely to take their children to hospital^27^. Since the beginning of the current decade, treatment of malaria has been made free for under-5 by the government, gradually increasing the number of under-5 consultations and hence the number of diagnosed malaria cases. Furthermore, the reporting system has been progressively improved, with the completeness rate of reports ranging from 79 to 87% between 2014 and 2018, resulting in an increase in the number of reported cases of malaria^26^.

In addition, the ineffective use of preventive methods such as mosquito bed nets may also be a plausible explanation, as previously reported in other sub-Saharan African countries^26,28,29^. Even though ownership has increased significantly (2% in 2004, 36% in 2011 and 73% in 2018), mosquito bed net usage is less effective in the country. According to the results of the 2018 Demographic and Health Survey (DHS), only 60% of the under-5 slept under a mosquito bed net the day before the survey^30^. The reasons for low mosquito bed net use reported in most surveys conducted in Cameroon are: heat-related discomfort, lack of access to bed nets and the perception of a low Anopheles density by the population^26^.

Our findings suggest heterogeneity within a region in terms of malaria incidence. This has already been reported in previous studies in Cameroon, and was hypothesised to be related to the variability, within the same region, of environmental factors influencing mosquito proliferation^31,32^. It has been suggested that the proliferation of female Anopheles is high during the rainy seasons because of the optimal temperature for their growth (16–34 °C) and the presence of breathing areas^32,33^. As the climate and vegetation vary greatly across regions of the country and the period of the year, the reproductive conditions for mosquitos are variable in time and space, and so does the incidence. Similarly, seasonality and malaria risk are very heterogeneous in time and space, probably for similar reasons, as in several sub-Saharan African countries^34,35^.

Results of the current study call for area-specific malaria prevention measures for under-5 in Cameroon, considering the season of the year and environmental conditions. More attention in preventive and therapeutic interventions should be given to the equatorial zone, especially to the East region which has most of the health districts at high risk of malaria. They also call for intensified efforts to reduce the burden of malaria in under-5 in Cameroon, to curb the trend of this life-threatening disease and to reach the country goal of malaria burden reduction.

Although the conclusions of this study are based on rigorous statistical analysis, the results must be interpreted in the context of some drawbacks. The population at risk was derived from the estimate made by the national institute of statistics based on the 2005 census and may be slightly different from the real population living in the different areas. Moreover, covariates that measure malaria interventions were not introduced into the Bayesian model, because of difficulties to synthetized them given the data in hand. Hence a variance in risk that may be due to those interventions was not captured. Furthermore, available data did not allow for a distinction between the proportion of cases diagnosed by microscopy or RDT, thus precluding any adjustment for differences in diagnostic technique. A more complete picture of both morbidity and mortality associated with malaria during the study period would have been provided by the inclusion of spatio-temporal analysis of mortality data. Nevertheless, this study is the first conducted on a time-series of routinely collected malaria data in under-5 living in Cameroon, adjusted according to the WHO recommendations. It is also the first to assess the seasonal variation of malaria incidence based on a robust and updated method of time series decomposition and to produce, using a Bayesian model, a map of malaria risk in under-5 in Cameroon.

## Conclusion

The analysis of the incidence of symptomatic malaria in children under-5 between 2012 and 2018 in Cameroon shows an overall upward trend at the national level, a substantial variation in the malaria trend, seasonality, and a variable pattern of malaria risk from one year to another, suggesting that the incidence of malaria in children under-5 in Cameroon is heterogeneous and varies between health districts and seasons of the year. It is therefore crucial to adapt malaria preventive measures to the specificities of each health district, considering seasonal variations in the incidence of this disease.

## Supplementary Information

Supplementary Information.

## Data Availability

The malaria count and all the demographic data can be obtained from the Cameroon Malaria Control Program, and all the data analysed in this study are available from the corresponding author under request and approval of the Cameroon NMCP. Health-seeking behaviour can be obtained from the DHS and UNICEF program. More details on data and statistical analyses can be found in the additional file.
