# Supplementary material for: Fine scale analysis of malaria incidence in under-5: hierarchical Bayesian spatio-temporal modelling of routinely collected malaria data between 2012–2018 in Cameroon
Source: Sci Rep. 2021 Jun 1;11:11408. doi: 10.1038/s41598-021-90997-8 (PMC8169670; doi:10.1038/s41598-021-90997-8)
Supplement: Supplementary file 1 — Supplementary Information. [file 41598_2021_90997_MOESM1_ESM.pdf]

Fine scale analysis of malaria incidence in under-5: Hierarchical Bayesian spatio-temporal modelling of routinely collected malaria data between 2012-2018 in Cameroun

Celestin Danwang[1*], Élie Khalil[1], Dorothy Achu[2], Marcelin Ateba[2], Moïse Abomabo[2], Jacob Souopgui[3], Mathilde De Keukeleire[1], Annie Robert[1]

APPENDIX

Supplementary Figures

Supplementary Fig. 1. Division of the country in regions and health districts. (Source: Ministry of public Health of Cameroon, URL : https://dhis-minsante-cm.org/portal/) 2
Supplementary Fig. 2. Correlation between Incident-cases, rainfall, temperature and vegetation index 4
Supplementary Fig. 3. Seasonal and trend decomposition of national malaria incident rate 6
Supplementary Fig. 4. Structured spatial effect of the final model with and without covariates. The map was generated with tmap package of R software version 4.0.2 (URL: https://cran.r-project.org/web/packages/tmap/vignettes/tmap-getstarted.html). 10
Supplementary Fig. 5. Unstructured effect of the frailty and the final model with covariates. The map was generated with tmap package of R software version 4.0.2 (URL: https://cran.r-project.org/web/packages/tmap/vignettes/tmap-getstarted.html). 11
Supplementary Fig. 6. Density plot of the random effect term of the frailty model 12
Supplementary Fig. 7. Density plot of the unstructured random effect term of the final model without covariates 12
Supplementary Fig. 8. Density plot of the unstructured random effect term of the final model with covariates 13
Supplementary Fig. 9. Density plot of the structured random (CAR) effect term of the final model without covariates 14
Supplementary Fig. 10. Density plot of the structured random (CAR) effect term of the final model with covariates 14
Supplementary Fig. 11. Exceedance probability (RR>1) risk map. The map was generated with tmap package of R software version 4.0.2 (URL: https://cran.r-project.org/web/packages/tmap/vignettes/tmap-getstarted.html). 15

Supplementary Tables

Supplementary table 1. Data source 3
Supplementary table 2. Summary results of the Mann-Kendall test and the seasonal-trend decomposition 5
Supplementary table 3. Moran's I statistic and P-value 7
Supplementary table 4. DIC of Bayesian models 8
Supplementary table 5. Fixed effects of the Bayesian spatiotemporal model 9

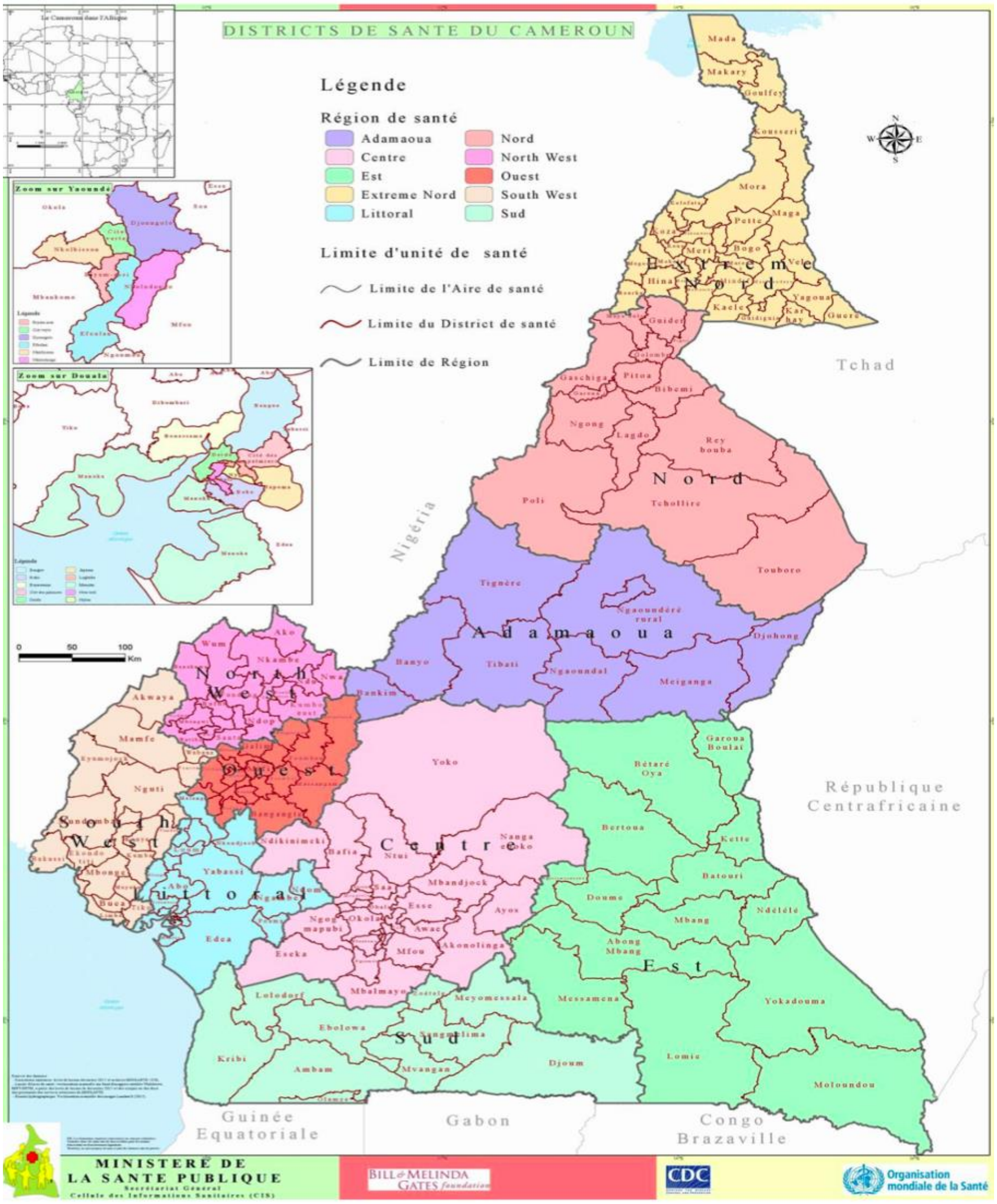

NB: Centre without Yaoundé corresponds to the administrative region of the centre without the health districts recognized as belonging to the city of Yaoundé according to the division of the Ministry of Health. The same definition applies to the littoral without Douala.

Supplementary Fig. 1. Division of the country in regions and health districts. (Source: Ministry of public Health of Cameroon, URL : https://dhis-minsante-cm.org/portal/)

Supplementary table 1. Data source

Data	Scale	Level	Period	Source
Malaria Counts	Health district	Monthly	2011-2018	NMCP
Under 5 years population*	Health district	Yearly	2011-2018	National Institute of statistics<br>(http://www.statistics-cameroon.org)
Health seeking behaviour	National	Yearly	2011 and 2018<br><br>2014	Demographic and health surveys (https://dhsprogram.com)<br>MICS (https://mics.unicef.org/surveys)
Temperature	Health district	Monthly	2011-2018	WorldClim<br>(https://worldclim.org)
NDVI	Health district	1km	2011-2018	USGS (https://modis.gsfc.nasa.gov/data/dataprod/mod13.php)
Rainfall	Health district	Monthly	2011-2018	WorldClim<br>(https://worldclim.org)

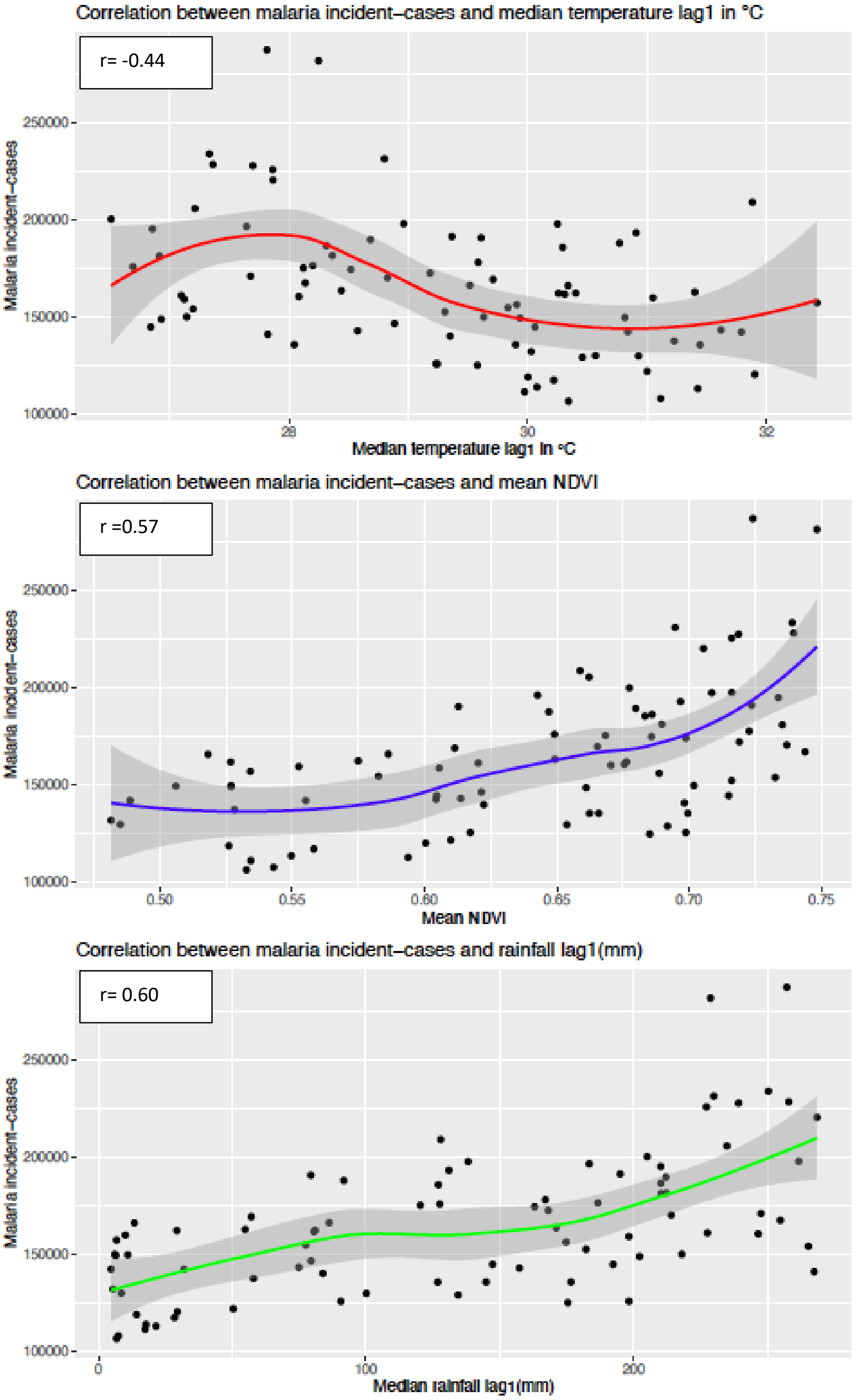

NB: the temperature was the median value of the maximal temperature

Supplementary Fig. 2. Correlation between Incident-cases, rainfall, temperature and vegetation index

Supplementary table 2. Summary results of the Mann-Kendall test and the seasonal-trend decomposition

Region	P-value of the Mann Kendall test	Overall Trend	Seasonality (Number of annual peaks)
Adamaoua	0.007*	Increased	Two
Centre (without Yaoundé)	0.02*	Increased	Two
Douala-Littoral	0.76	No monotonic trend	Four
East	0.04*	Increased	Two
Far North	0.22	No monotonic trend	One
Littoral [without Douala]	0.13	No monotonic trend	Two
North	0.04*	Decreased	Two
North-West	0.13	No monotonic trend	Two
South-West	0.37	No monotonic trend	Three
South	0.07*	Increased	Two
West	0.76	No monotonic trend	Two
Yaoundé-Centre	0.37	No monotonic trend	Two

**significant.*

NB: The Mann-Kendall test was performed on the number of malaria cases and the seasonal decomposition on the incidence per month.

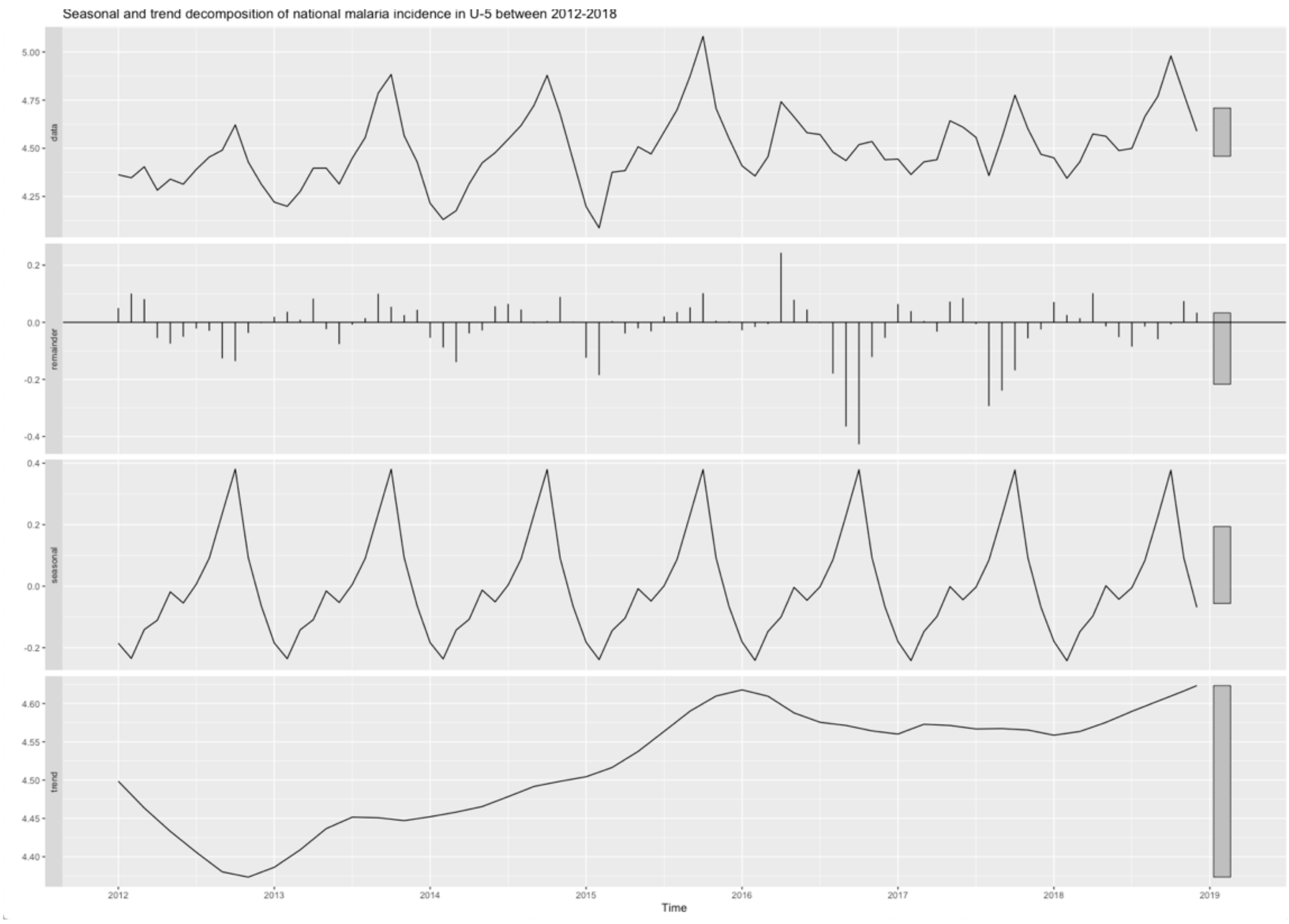

Supplementary Fig. 3. Seasonal and trend decomposition of national malaria incident rate

Supplementary table 3. Moran's I statistic and P-value

	Value of the Moran'I statistic (P-value)						
Year	2012	2013	2014	2015	2016	2017	2018
January	0.11 (0.008)	0.18 (<0.001)	0.13 (0.002)	0.17 (<0.001)	0.31 (<0.001)	0.34 (<0.001)	0.33 (<0.001)
February	0.12 (0.005)	0.15 (0.001)	0.15 (<0.001)	0.23 (<0.001)	0.30 (<0.001)	0.36 (<0.001)	0.31 (<0.001)
March	0.12 (0.006)	0.19 (<0.001)	0.14 (<0.001)	0.28 (<0.001)	0.28 (<0.001)	0.37 (<0.001)	0.38 (<0.001)
April	0.20 (<0.001)	0.25 (<0.001)	0.24 (<0.001)	0.27 (<0.001)	0.36 (<0.001)	0.41 (<0.001)	0.41 (<0.001)
May	0.16 (<0.001)	0.12 (0.005)	0.33 (<0.001)	0.36 (<0.001)	0.42 (<0.001)	0.46 (<0.001)	0.43 (<0.001)
June	0.22 (<0.001)	0.20 (<0.001)	0.27 (<0.001)	0.32 (<0.001)	0.41 (<0.001)	0.46 (<0.001)	0.37 (<0.001)
July	0.37 (<0.001)	0.33 (<0.001)	0.35 (<0.001)	0.31 (<0.001)	0.37 (<0.001)	0.34 (<0.001)	0.33 (<0.001)
August	0.45 (<0.001)	0.53 (<0.001)	0.49 (<0.001)	0.46 (<0.001)	0.44 (<0.001)	0.34 (<0.001)	0.46 (<0.001)
September	0.31 (<0.001)	0.50 (<0.001)	0.53 (<0.001)	0.48 (<0.001)	0.43 (<0.001)	0.40 (<0.001)	0.45 (<0.001)
October	0.32 (<0.001)	0.50 (<0.001)	0.50 (<0.001)	0.51 (<0.001)	0.40 (<0.001)	0.37 (<0.001)	0.47 (<0.001)
November	0.25 (<0.001)	0.26 (<0.001)	0.35 (<0.001)	0.39 (<0.001)	0.34 (<0.001)	0.31 (<0.001)	0.36 (<0.001)
December	0.14 (<0.001)	0.11 (0.006)	0.19 (<0.001)	0.22 (<0.001)	0.34 (<0.001)	0.28 (<0.001)	0.28 (<0.001)

Supplementary table 4. DIC of Bayesian models

Model	DIC of the Frailty model	DIC of the model without covariates	DIC of the model with covariates
Empty model (IID)	17,282.46	-	-
Model (BYM)	-	17,282	17,275
Uncorrelated time (Date IID)	-	17,343	17,348
1st order random walk correlated time (Date RW1)	-	17,324	17,339
(Date RW1) and (Date IID)	-	17,324	17,351
(Date RW1) and space-time interaction term (time intx)	-	17,451	17,515
(Date RW1) and (Date IID) and (Date intx)	-	17,457	17,520

DIC: Deviance information criterion

Supplementary table 5. Fixed effects of the Bayesian spatiotemporal model

Indicator	Frailty model<br><br>Posterior mean (95% CrI)	Final model without covariates<br><br>Posterior mean (95% CrI)	Final model with covariates<br><br>Posterior mean (95% CrI)
**Fixed effects**			
Intercept	0.91 (0.82;1.00)	0.91 (0.87;0.94)	1.21 (0.98;1.49)
Rainfall (mm/100)			0.91 (0.90;0.92)
NDVI			3.41 (3.13; 3.73)
Temperature (ºC)			0.97 (0.97; 0.98)
DIC	17,283	17,282	17,275

NDVI: Normalized Difference Vegetation Index; DIC: Deviance information criterion

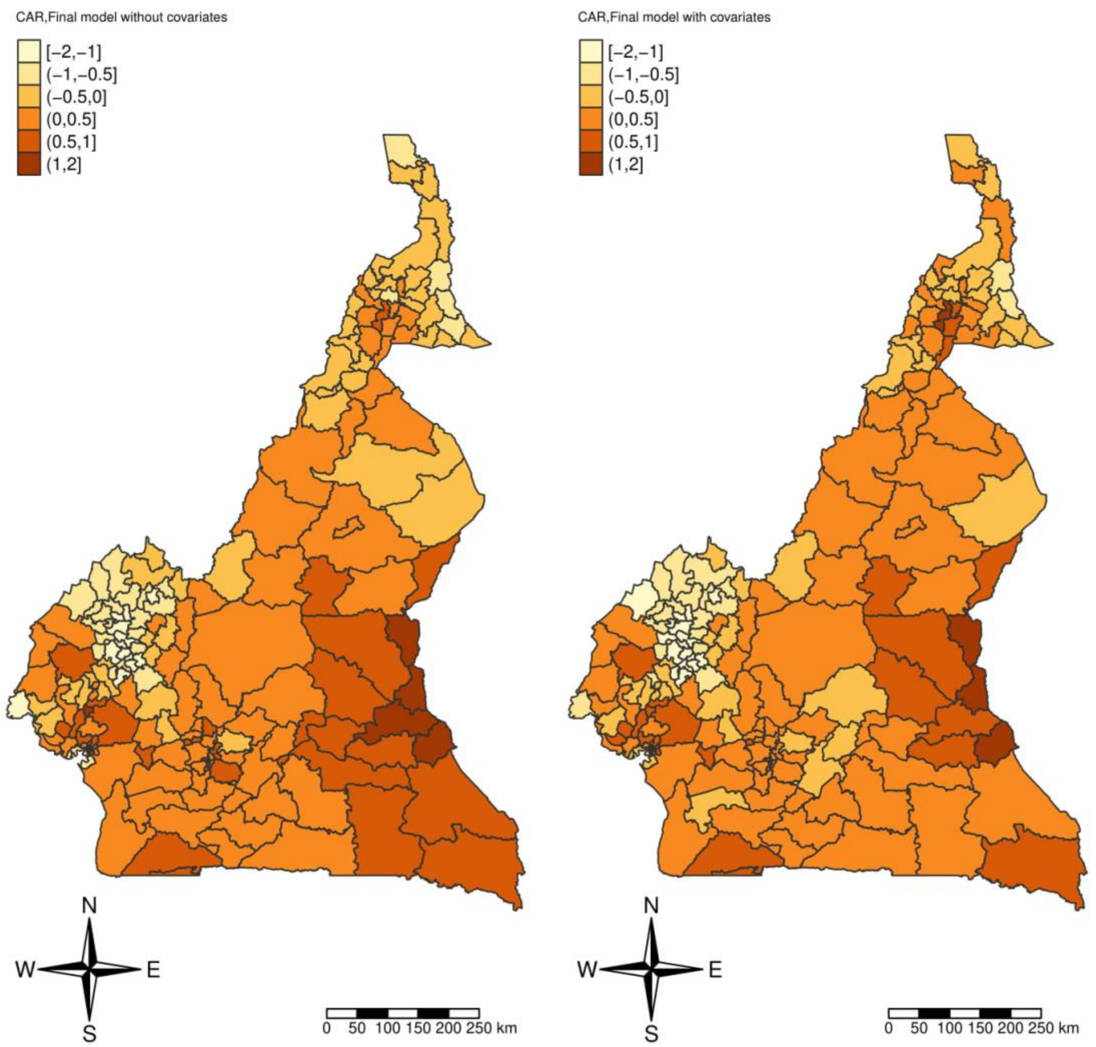

Supplementary Fig. 4. Structured spatial effect of the final model with and without covariates. The map was generated with tmap package of R software version 4.0.2 (URL: https://cran.r-project.org/web/packages/tmap/vignettes/tmap-getstarted.html).

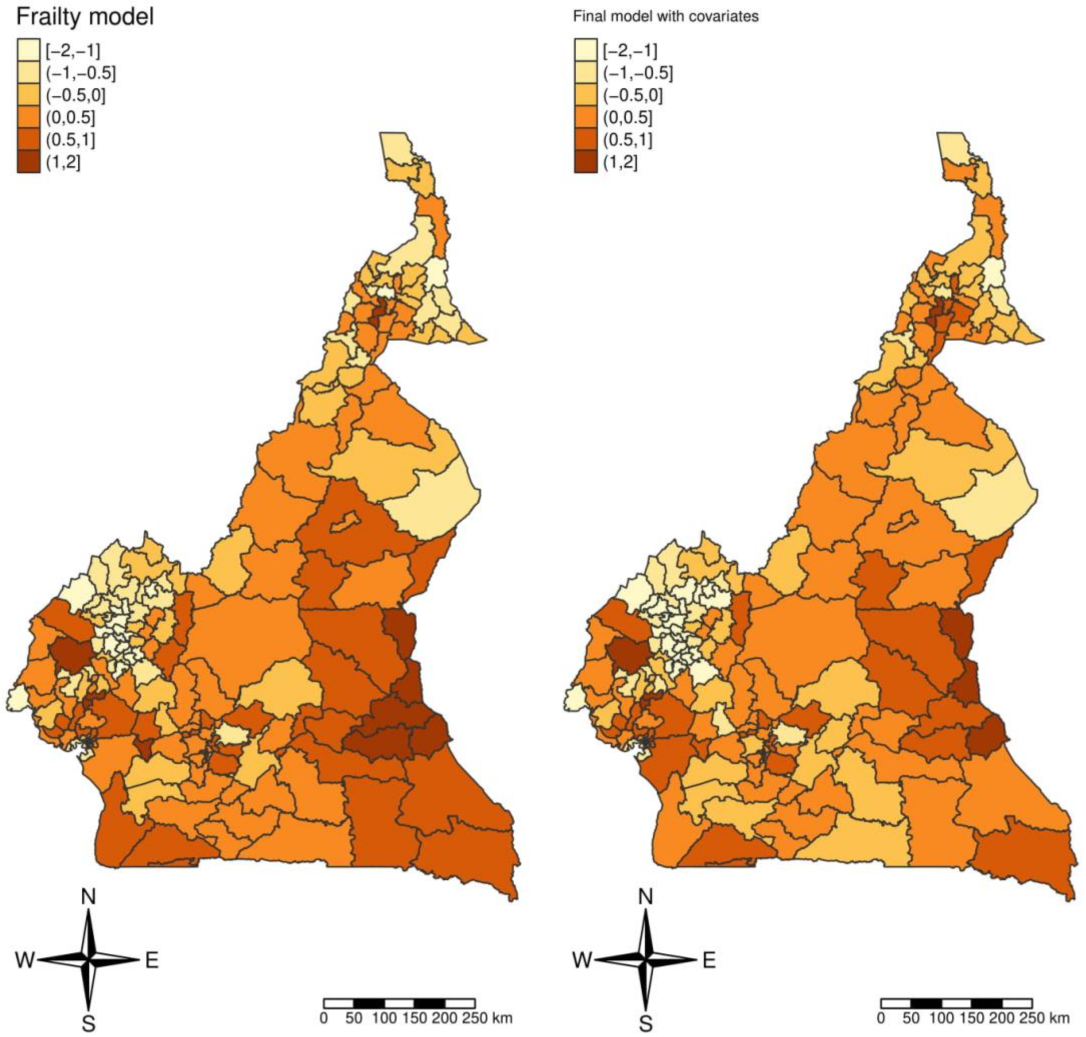

Supplementary Fig. 5. Unstructured effect of the frailty and the final model with covariates. The map was generated with tmap package of R software version 4.0.2 (URL: https://cran.r-project.org/web/packages/tmap/vignettes/tmap-getstarted.html).

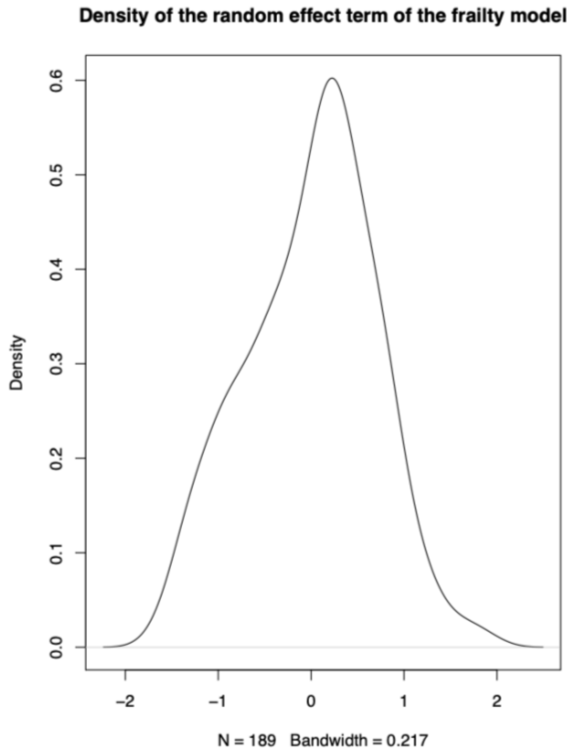

Supplementary Fig. 6. Density plot of the random effect term of the frailty model

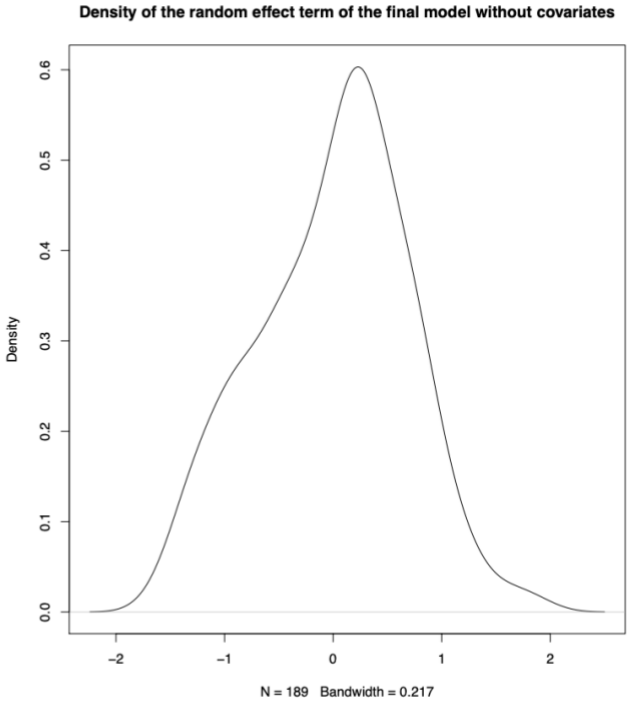

Supplementary Fig. 7. Density plot of the unstructured random effect term of the final model without covariates

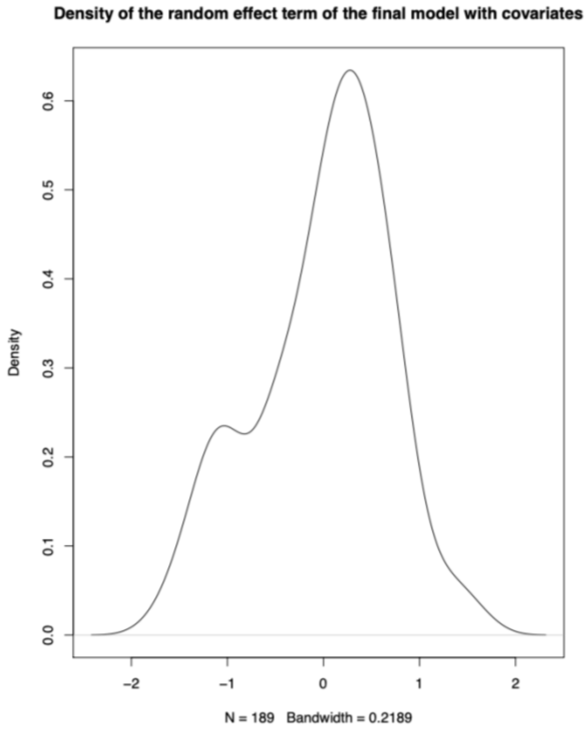

Supplementary Fig. 8. Density plot of the unstructured random effect term of the final model with covariates

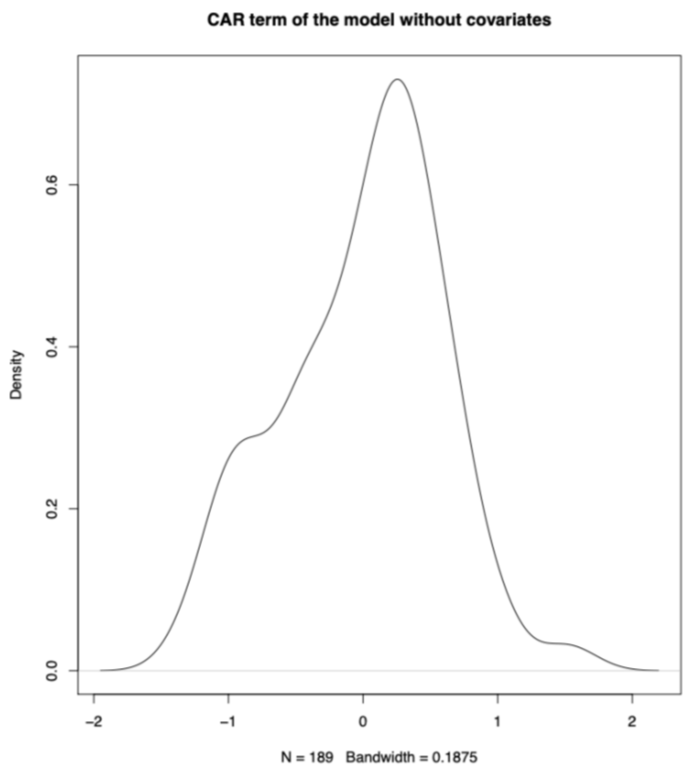

Supplementary Fig. 9. Density plot of the structured random (CAR) effect term of the final model without covariates

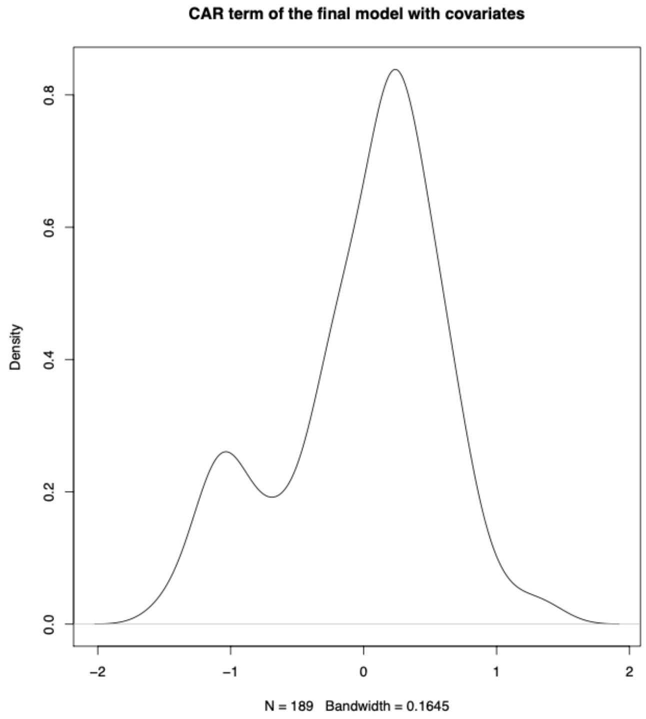

Supplementary Fig. 10. Density plot of the structured random (CAR) effect term of the final model with covariates

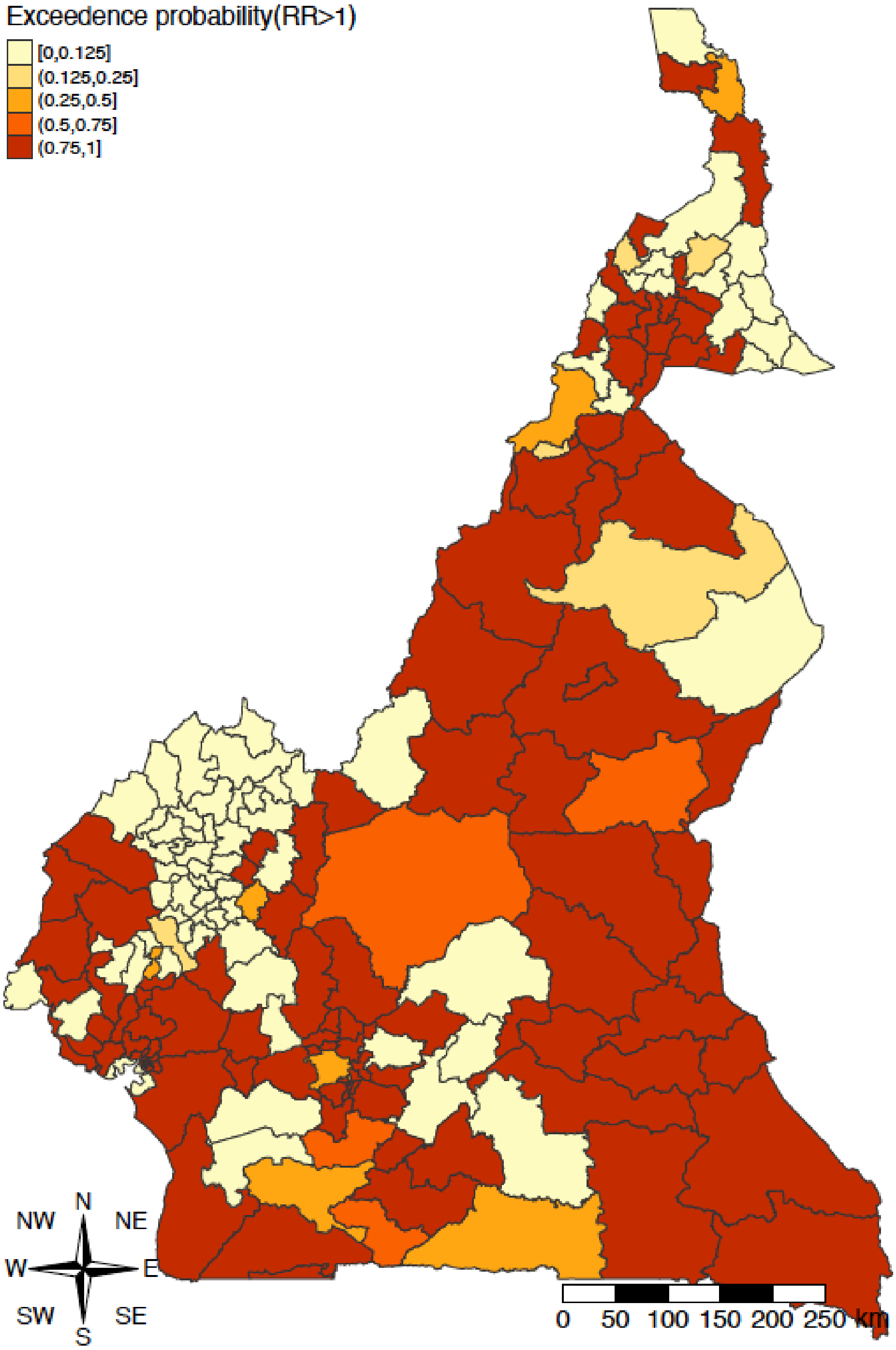

Supplementary Fig. 11. Exceedance probability (RR>1) risk map. The map was generated with tmap package of R software version 4.0.2 (URL: https://cran.r-project.org/web/packages/tmap/vignettes/tmap-getstarted.html).
